# Mediators of maternal intergenerational epigenetic inheritance in mammals

**DOI:** 10.1080/17501911.2025.2525749

**Published:** 2025-07-03

**Authors:** Christian Belton, Gavin Kelsey

**Affiliations:** a Epigenetics Programme, The Babraham Institute, Cambridge, UK; b Loke Centre for Trophoblast Research, University of Cambridge, Cambridge, UK; c Wellcome-MRC Institute of Metabolic Science-Metabolic Research Laboratories, Cambridge, UK

**Keywords:** Epigenetic inheritance, imprinting, metastable epialleles, maternal effects, germline

## Abstract

Experimental models and epidemiological data suggest that environmental factors, for example, adverse nutrition prior to conception, can lead to phenotypes in offspring of exposed parents in the absence of continued exposure. As a result these phenotypes have been described as epigentically inherited. The mechanistic basis for such phenomena has not been established in most cases. In this review, we consider possible contributing mechanisms for environmentaly induced epigenetic inheritance, with a focus on maternally transmitted effects and by comparing to paradigms of epigenetic inheritance with a clear mechanistic understanding. Genomic imprinting has provided an important conceptual framework for how the epigenetic states of parental germlines can determine allelic expression in offspring, yet, generally speaking, imprinted genes appear resilient to epigenetic disruption from altered parental environments. Metastable epialleles are environmentally sensitive and variably expressed loci that can impact organism phenotype, but the nature of any epigenetic marker at these loci transferred to offspring is unclear. Studies of examples across these forms of epigenetic inheritance show predominant effects are mediated by oocyte factors involved inreprogramming of the genome post-fertilization, rather than direct effects on gametic DNA methylation, with the exception of genomic imprinting. The potential contribution of additional oocyte chromatin features to the specific liability of phenotypic effector genes and their potential to persist through this reprogramming, however, remains to be investigated.

## Plain Language Summary

Experience of parents can change traits in their offspring and this doesn’t require changes in DNA. It’s not really understood what causes this in mammals and it’s important because it could change medical and veterinary practices. Certain traits are only inherited from a male parent, and others from a female parent, and the causes for this are better understood. In this review, we compare what is known about these types of DNA independent inheritance. We find causes they have in common happen after the egg has been fertilized. This makes sense because lots of information in embryos is wiped at this point. But when inheritance is the result of experience, because of this information wipe, no one has really looked earlier for causes. It might be that the causes are there, which is exciting because they would have to be completely different from the currently known causes of inheritance.

## 1. Introduction

### 1.1. DNA sequence independent effects on offspring phenotype

It has been well demonstrated by classical experiments that rates of change in organism phenotype can be influenced via both DNA sequence dependent and independent mechanisms [1]. DNA independent phenotypes can also arise in offspring as the result of parental phenotype, and examples of this have been reviewed extensively elsewhere [2–7]. The absence of evidence for genetic inheritance of these offspring phenotypes seems to be widely considered sufficient to classify them as epigenetically inherited. However, until the molecular mechanism of transmission is established, cryptic genetic influences on this heritability cannot be fully ruled out. Variations in patterns of epigenetic inheritance between phenotypes indicate differences in their molecular mechanism of transmission. Mechanisms mediating the persistence of phenotypes intergenerationally when an individual, or their parental germline, is directly subjected to conditions that lead to an offspring phenotype will have different requirements to those persisting beyond this, or transgenerationally [8]. Additionally, phenotypes that show different distributions of inheritance in each sex of parent may be mediated differently within each sex of parental germline or influenced by differential contribution of each parent to gestational environment [9].

In this review, we focus on intergenerational inheritance through the maternal germline of mammals, comparing known factors required for transmission and the genes impacted by forms of epigenetic inheritance with varying levels of experimental tractability. In doing so, we seek to assess to what extent and at what developmental stage mediators can be shared in common between these forms and whether experimental methods applied to one form can be applied usefully to another.

### 1.2. Life-cycle essential, gene-linked epigenetic inheritance – genomic imprinting

Phenotypes where causal alleles are transmitted via both sexes, but expression only occurs when inherited via a specific parent, have long been recognized in mammals [10,11]. The signal specifying the parent-of-origin of these alleles must be epigenetically inherited, intergenerationally reset, and then newly established via a process termed genomic imprinting. Lethality and non-equivalence of phenotypes resulting from forced uniparental inheritance of genomes underlines the importance of genomic imprinting in the mammalian life-cycle [12–14]. Ability to detect the parent-of-origin effects of mutations via the linkage of phenotypes to uniparental inheritance of genetic loci has enabled genetic screens in mice to identify many imprinted genes [15]. The conservation of these genes, along with recognition of parent-of-origin inheritance pattern, has also enabled identification of the phenomenon as a cause of human genetic disorders [16]. Genetic experiments have also enabled testing of theoretical mediators both in the germline and post-fertilization, as well as the mapping of imprinting control regions (ICRs), elements required in *cis* for imprinted gene expression [15]. This has led to unrivaled understanding of the molecular mediators of imprinting and its use as a paradigm for other epigenetic phenomena [17,18].

### 1.3. Life-cycle dispensable, gene-linked epigenetic inheritance – MEs

Mutations in mice can lead to phenotypic variation between genetically identical individuals that are invariant in wild-type animals, and examples exist where the distribution of phenotypes in offspring is correlated with the level of expression in parents, indicating they are subject to epigenetic inheritance [19]. The most well-known of these is the *agouti* locus (*A*) in mice. Mice possessing a mutated allele of the locus (*A*^*vy*^) produce genetically identical offspring with varying coat color [20]. The variable pattern of coat color in offspring of these mice, however, is not completely random, with yellow mothers producing a greater proportion of yellow offspring than those that are pseudoagouti (brown) [21]. These variable phenotypes, and associated alleles, are referred to as “metastable,” a term originally used to define variable phenotypes resulting from paramutation in plants [22]. The most well-characterized examples of metastable phenotypes in mice both result from insertions of intracisternal A-particle (IAP) endogenous retroviral elements (ERVs) into loci with dosage-dependent effects on phenotype [20,23]. Phenotypic expression in individuals is correlated with the levels of DNA methylation, a chemical modification of DNA, at the associated IAPs [24–26]. The presence or absence of DNA methylation at a locus, not solely determined by its DNA sequence, is capable of influencing gene expression and is heritable through cell divisions, leading to it being referred to as an epigenetic mark [27]. Consistent DNA methylation at these alleles across the tissues of an individual indicates that a pattern was established early in development which has been subsequently maintained and distinguishes them from tissue variably methylated regions [24,28]. The link between inter-individual variation of an epigenetic modification and the distribution of metastable phenotypes defines these loci as metastable epialleles (MEs). Despite strong genetic linkage, MEs lack a universal phenotype when disrupted. This has forced screens identifying naturally occurring MEs to focus on assumed features, such as association with ERVs, tissue uniformity in inter-individually variably expressed genes, and inter-individual variation in DNA methylation across large genetically diverse cohorts in human studies [29]. In mice, when tested, heritability of methylation state at ERVs identified by these screens is mostly absent and weak when detectable [30]. Nonetheless, features of MEs with verified epigenetic inheritance have been studied, allowing for the relation to other forms of epigenetic inheritance.

### 1.4. Life-cycle dispensable, gene-unlinked epigenetic inheritance – environmentally induced effects

A huge variety of pharmacological, dietary, psychological, and behavioral interventions modeling environmental exposures lead to epigenetically heritable phenotypes across spectra of model organisms (reviewed by others here [4]). Parental diet is the most well studied of these, with epigenetic effects observable across animal species [31–34]. Offspring phenotypes caused by these exposures are not always analogous to those that developed in parents, are often highly variable and, counterintuitively from an evolutionary perspective, are deleterious [7,35]. In mammals, the inheritance of environmentally induced phenotypes often lacks demonstrated genetic linkage and variability in phenotypes of offspring makes genetic screening for modifiers of such effects near impossible. This has led studies to focus on high-throughput assays or candidate gene approaches to detect changes at the molecular level in phenotypically relevant tissues following interventions, and ascribing causality to observed changes is difficult due to the pleiotropy inherent to many environmentally induced phenotypes. Providing evidence of selective pressure for environmentally induced epigenetic inheritance is essential for assessing its evolutionary importance, with competing arguments differing largely on the extent to which this is judged to be demonstrated [36–38]. It is also important practically, informing interventions and interpretation of molecular information in a medical and public health context [39,40]. For this selective pressure to occur, mechanisms must exist to ensure inheritance, and recent work has focused on the study of interactions between environmental interventions and mediators of gene-linked forms of epigenetic inheritance to uncover these.

## 2. Main body

### 2.1. Mediators across forms of epigenetic inheritance in the maternal germline

The theoretical potential for molecular changes leading to inheritance is much greater in oocytes than sperm. Cytoplasmic factors in the early embryo are predominantly oocyte derived [41,42]. These in turn contribute to mechanisms that actively reprogram the paternal pronucleus post-fertilization, which are dependent on maternally derived histones and chaperones, such as HIRA, and DNA demethylases, such as TET3 [43–45]. Importantly, for the potential inheritance caused by environmental effects, the window in which parental environment can influence DNA methylation establishment is also greater in oocytes, with completion achieved postnatally compared to prenatally in sperm [46–48]. Most histone modifications accumulated during the initial stages of spermatogenesis are also stripped from the mature sperm genome during the transition from round spermatids [49–51].

Factors present in the maternal germline have been shown to be sufficient for epigenetic inheritance for examples from all classes of tractability. The demonstration of failure of development of parthenogenetic and gynogenetic embryos [12,13] together with the observation that imprinted genes that are not expressed in parthenotes generated via transfer of nuclei from mature oocytes are expressed in parthenotes generated from immature oocytes, reveal that oocyte nuclear factors are both necessary and sufficient for establishment of imprints in the very early embryo [52]. The skewed distribution of coat colors expected from offspring of yellow *A*^*vy*^ dams is observed even when embryos generated using yellow *A*^*vy*^ oocytes are transferred to pseudoagouti surrogates [25]. Intergenerationally inherited environmentally induced phenotypes can also be mediated, if not exclusively, by factors in the maternal germline, with both maternal and paternal effects of high fat diet (HFD) exposure being observed in offspring produced via *in vitro* fertilization, with subtle differences in phenotype depending on parent exposed [33].

Work on uncovering the specific nuclear factors necessary for the establishment of imprints in the maternal germline reveals that chromatin-modifying enzymes, and the *cis* elements that direct them, are essential. Maternal loss of *trans*-acting factors facilitating DNA methylation in the oocyte cause loss of imprinted gene expression post-fertilization [53,54]. Maternal germline-specific *cis* elements (oocyte transcription start sites) facilitate DNA methylation of germline differentially DNA methylated regions (gDMRs) in both mice and humans [55,56]. This facilitation also requires the methylation of histone 3 lysine 36 (H3K36me3) by oocyte-derived SETD2 enzyme [57]. High-throughput characterization of germ cell DNA methylomes has confirmed that many gDMRs overlap with previously mentioned, genetically required, ICRs [47]. In addition to DNA methylation, several genes in rodents have also been discovered to be subject to paternal allele-specific expression exclusively in placental lineages, are marked by H3K27me3, and require maternal EED, a component of the PRC2 complex that catalyzes the addition of this mark, for maternal allele silencing post-fertilization [58–60].

The inheritance of the ME state is dependent on parental sex [21,30] and in some examples, such as *Axin*^*fu*^, exclusive maternal inheritance can be induced through changes in genetic backgrounds [24]. This indicates *trans-*acting factors are capable of influencing germline mediated inheritance, nonetheless, the investigation of the germline epigenetic state of the loci, and their dependencies has been limited. DNA methylation of the *A*^*vy*^ locus in oocytes of an individual is consistent with that present globally, leaving open the possibility that the mark mediates the inheritance of skewed distribution in offspring [61]. Indeed, targeted modification of the locus in oocytes has been reported to lead to a skewed coat color distribution consistent with the methylation level induced, but it cannot be ruled out that this is a result of targeting of residual sgRNA and dCas9 fusion proteins to the locus later in development [62]. Putative human MEs seem to lack such a chromatin signal and do not show inter-individual variation in oocytes, suggesting that this is acquired later in development [63].

When searching for a signal in the germline in models of environmentally induced inheritance using treated and untreated comparisons, establishing oocyte factors as causal of an offspring phenotype can be challenging due to the inherent interconnectivity between maternal and fetal physiology [9,64]. This has led many studies into the mechanisms of environmental epigenetic inheritance to focus on the paternal line (reviewed recently here [65–67]). It is possible, though, to test for the presence and impact of candidate mediators shown to be relevant in more tractable forms of epigenetic inheritance. Chromatin modifications (including DNA methylation) have demonstrated biological significance and the feasibility of their modification in response to environmental exposures in oocytes [68–70]. Alterations in DNA methylation and other chromatin modifications have been observed in interventions modeling environmental exposures. For example, associations have been reported between changes in DNA methylation at the *Lep* and *Ppar-α* promoters in F0 oocytes and maternal HFD that plausibly contribute to the observed phenotype, with some correlation observed with the methylation status and gene expression at the same loci in F1 livers [71]. High-throughput interrogations of DNA methylation and gene expression in multiple maternal HFD F1 tissues find thousands of differently methylated regions (DMRs) and differentially expressed genes [72,73]. Connecting these changes to causal germline effects, is difficult. Despite the relative consistency between gene expression changes and metabolic phenotype, there is little overlap between DNA methylation changes in different tissues and high intra-individual variation. This implies that what is being measured is accumulated acquired effects, rather than a directly inherited oocyte state, though the lack of a study measuring both F0 oocytes and F1 tissues in the same model makes it difficult to judge if this is universally the case.

### 2.2. Maternal determination of acquired effects post-fertilization

Resetting the marks determining the differential expression of each generation is a requirement for a parent-of-origin effect. Previous parental patterns of DNA methylation are wiped genome-wide during gametogenesis and then reestablishment over gDMRs during gametogenesis prior to fertilization [55,56,74,75]. There is a second genome-wide erasure of parental chromatin marks post-fertilization and maternal contribution of a variety of factors stored in oocyte cytoplasm is required to prevent acquired loss of DNA methylation at gDMRs. Maternal DNA methylation maintenance enzyme DNMT1 prevents passive loss of gDMR methylation through early cell divisions [76]. STELLA/PGC7/DPPA3 is required for the retention of maternal genome methylation through suppression of the active DNA demethylase enzyme TET3 enzyme [77,78]. In addition, the KRAB zinc-finger proteins ZFP57 and ZFP445 bind specifically to gDMRs with their interacting partner the E3-ligase scaffold protein TRIM28, a transcriptional co-repression, with *Zfp57* and *Trim28* maternally required for gDMR protection [79–81].

The loss of non-imprinted marks on chromatin during post-fertilization erasure, which has been directly assayed in the case of *A*^*vy*^ [61], is part of the reasoning behind the previously mentioned lack of investigation of oocyte chromatin features in the study of non-imprinted forms of epigenetic inheritance. In contrast to imprinted genes, acquired gain of chromatin marks is required for epigenetic inheritance in these other classes of tractability. This has been investigated most extensively in a context comparable to MEs via an unbiased ENU mutagenesis screen for paternally contributed dominant negative modifiers of variable epigenetic transgene silencing, which has identified several known chromatin-modifying enzymes required for chromatin establishment and maintenance [82]. Many of these factors, such as DNMT1 and TRIM28, are commonly required for imprinted inheritance, and although their impact on coat color phenotype was assessed on paternal zygotic contribution, phenotypes for the *Trim28*^*D9/+*^ mutants are present with both maternal and paternal transmission [83].

As previously discussed, chromatin components of the molecular changes associated with dietary interventions are also more likely acquired effects, leading to the investigation of oocyte cytoplasmic factors as possible mediators of inheritance. Comparative proteomics of metaphase II (MII) oocytes from HFD female mice observes a reduction in the previously mentioned STELLA protein. DNA from zygotes produced by IVF of HFD MII oocytes is, concordant with the known functions of STELLA, hypomethylated, and this is rescuable, along with aspects of the F1 phenotype, by injection of *Dppa3* mRNA. The methylation phenotype and partially penetrant failure to reach the blastocyst stage is also rescuable by siRNA mediated depletion of *Tet3*, indicating that the post-fertilization role of STELLA, and not its other reported function in oocyte chromatin establishment, is instrumental in phenotype [84,85]. A more recent study, using a drug-induced hyperglycemia model of epigenetically transmitted glucose intolerance, observed that the abundance of *Tet3* mRNA in oocytes was particularly sensitive to the maternal hyperglycemic environment. DNA methylation effects on the paternal alleles of plausible phenotypic genes are present in the target tissues of this model; with molecular and metabolic phenotypes both recapitulated in maternal knock-out of *Tet3 and* were rescuable on the injection of *Tet3* mRNA into oocytes from hyperglycemic mothers [86]. Early chromatin state has been shown not to solely be mediated through changes in the expression level of maternally contributed chromatin modifying enzymes. Mutation of the mitochondrial DNA polymerase POLG, which leads to an increase in mitochondrial genome mutation rate, is also causal of global DNA hypomethylation in nuclear oocyte genomes, reduced removal of DNA methylation post-fertilization, and late gestational developmental defects [87]. Importantly, the F1 post-fertilization defects can be rescued by supplementing *in vitro* embryo culture media with α-ketoglutarate, a metabolic co-factor required with TET3 activity, revealing a role for such co-factors in the modulation of early embryo chromatin state [87,88].

Together, these studies focusing on early embryonic development suggest that chromatin is causally impacted by these interventions, in keeping with the idea that commonality could exist between the mediators maintaining the more tractable epigenetic inheritance and environmental effects. However, in contrast to imprinting, these chromatin perturbations are caused by changes in cytoplasmic maternal factors and only subsequently transduced to chromatin during zygotic reprogramming. The inheritance of oocyte chromatin perturbations during germ cell development is not necessary.

Bridging the gap between these chromatin effects and the F1 phenotype also remains difficult. When measured, there are only small changes in total CpG methylation at candidate promoters in the effector tissues of the phenotype, leaving an open question whether this is the result of incomplete penetrance of heritability between individual cells, with the small number of perturbed cells being sufficient to cause a phenotype, or whether early DNA methylation changes in these instances are indicative of perturbation in an uncharacterized less discrete mark of the locus. Disentangling these possibilities is challenging with evidence of causality between chromatin changes observed in gene expression lacking. It is also unclear to what extent the incomplete penetrance of dosage reduction in chromatin-modifying factors in individual MII oocytes contributes to variability in these F1 phenotypes. Study of cases where gene expression has been directly linked to changes in chromatin state and penertrance is tractable across development can help to distinguish between these possibilities.

### 2.3. Commonality between genes impacted by epigenetic inheritance mediated by different mechanisms

Speculation on the evolution of imprinting theorizes that subject gene action is especially sensitive to dosage, the phenotype on silencing of a parental allele thus being capable of being selected for [89]. This has been extensively experimentally supported, especially in the context of placental development, with mutants deleting or overexpressing given imprinted genes leading to opposite phenotypes with regards to fetal growth [90]. Although this dosage dependence allows imprinted genes to be plausible effectors for environmentally induced epigenetic phenotypes, which often cause small changes in expression, it could equally prevent them from being so, as they are feasibly less likely to be altered because of selection for particularly robust control. Indeed, resilience of oocyte gDMRs has been observed in response to both maternal HFD and low-protein diet (LPD) [71,91]. However, changes in imprinted gene expression do occur in the F1 of an undernutrition model, albeit at a frequency lower than that of bi-allelically expressed genes [92]. Complete loss of imprinting in the *Dlk1-Dio3* region causes pre-birth lethality; however, partial overexpression of the locus on the maternal allele leads to a subtler post-natal phenotype, with impaired brown adipose tissue (BAT) deposition. This supports the idea that manipulations of imprinted gene dosages more subtle than complete loss of maternal silencing are phenotypic [93,94]. Manipulation of expression for an imprinted gene within the same region, *Dio3os*, has also been shown to modulate BAT deposition in maternal HFD, providing hope that imprinted genes may be experimentally tractable effectors of environmental phenotypes [95].

The insertion of a luciferase reporter downstream of the endogenous *Dlk1* locus on the maternal allele reveals that loss-of-imprinting (LOI) in response to HFD is indeed variable in adult effector tissues of the intergenerational phenotype, rather than partially present uniformly throughout the animal, with interindividual variability also apparent in size and extent of effect [96]. Importantly, individuals with less LOI were less subject to the epigenetically inherited phenotype of the environmental perturbation, supporting the fact that partial penetrance of LOI contributes to phenotype. This variable effect of parental diet on LOI is also not restricted to maternally repressed genes, or exclusive to maternal HFD exposure, with *Cdkn1c* showing re-expression of the paternal allele on maternal exposure to LPD in a similar luciferase reporter mouse [97]. Use of the molecularly well-understood paradigm of imprinting also shows that discordance between physiological effects of intervention on parents and F1 phenotype, characteristic of epigenetically inherited traits, extends to the level of chromatin, with environmentally exposed animals having no observed LOI in adult tissues. However, DNA methylation of at least the *Dlk1* gDMR is unchanged in oocytes of HFD mothers, revealing that effector gene modulation is not always due to the inheritance of germline DNA methylation even in the case of imprinted genes.

ME controlled genes have also been reported to be influenced by environmental exposures, with proportions in offspring coat color altered when pregnant *A*^*vy*^ mothers are subjected to dietary or pharmacological changes in environment [28,98,99]. Hits from mouse screens used to identify MEs, however, show remarkable resilience in the face of environmental perturbations, akin to imprinted gene repression, hinting that these loci are protected from perturbation in the maternal germline, and robust establishment of interindividual variation post-fertilization may be selected for [100]. The possibility that the environment interacts with MEs more broadly has also led to efforts to uncover their existence and this effect in humans. Screens for loci with intra-individual variation in DNA methylation (not the inter-individual variation characteristic of murine MEs) within human populations in the Gambia have uncovered regions that seem to vary in methylation based on season of conception and correlated with changes in blood chemistry of mothers that arise due to differential nutritional intake [101,102]. Widening these screens to include individuals of different ethnicities and narrowing criteria for ME discovery to require consistency in methylation within tissue of common embryonic lineage, identifies MEs at a polymorphic imprinted gene that plausibly interact with the phenotypes observed in individuals conceived within a given season [103]. Candidate-based approaches screening for variability in DNA methylation in genes that could explain obesogenic phenotypes uncover a potential ME that also seems to vary depending on the season of conception [104]. Despite these associations between environment and putative ME methylation, it is hard to directly relate these human MEs to those observed in mice. Unlike *A*^*vy*^, the season of conception variable MEs normalizes as children mature, and methylation level is partially associated with genetic mutations [105]. This may be due to the laxer criteria on interindividual variation in methylation applied in humans. More recently, mouse screens have also reported tissue-specific variable methylation patterns, yet it remains to be seen if these possess phenotypic features[106]. Exactly how environmental exposures interact with the mediators for inheritance of ME phenotypes is unknown. Investigation into this is difficult, due to the variability of associated phenotypes, and controversy over whether the effects seen for exposures even in the archetypical ME in mouse, *A*^*vy*^, may not be detectable in larger samples [107].

## 3. Future perspectives

The comparison of the mediators between forms of epigenetic inheritance here highlights both the relative under investigation of germline mediators, especially on chromatin, in non-imprinted forms, and that when mediators are shared, they primarily act post-fertilization. When genes are influenced in multiple forms of inheritance, the potential for common mechanisms of regulation has only been tested in relation to gDMRs, which have been found to be unaffected by environmental exposures to parents.

The absence of evidence for chromatin mediators in the germline for other means of epigenetic inheritance does not mean they do not exist. Nuclear transfer experiments in germ cells, such as those carried out to demonstrate the need for genomic imprinting, have not been carried out to test which compartments of germ cells are required for other forms of epigenetic inheritance. It could be argued that cytoplasmic germ cell factors are more important outside of imprinting, due to post-fertilization erasure of chromatin marks. However, the lack of explanation of how such factors perpetuate themselves into later development means that, given what is known, they must lead to acquisition of chromatin marks. This in turn necessitates a means of transduction of signals between cytoplasmic and nuclear compartments, and it is not clear why this is favored as an explanation over the reverse requirement for germ cell chromatin mediators that are wiped during erasure to temporaily be transduced to a chromatin signal. The lack of fidelity in these transduction processes may explain the weakening of epigenetically inherited phenotypes across generations.

Another argument against germ cell or early developmental chromatin mediation is that it is hard to imagine how discrete modifications in a given cell, such as DNA methylation, lead to the intraindividual variability and interindividual variation associated with non-imprinted forms of epigenetic inheritance. Strikingly, a recent study ectopically inducing DNA methylation via transient mutagenesis of CpG islands has observed persistence of induced marks transgenerationally. Despite the absence of the parental DNA methylation in germ cells, the extent of DNA methylation present at the parental locus is recapitulated in the soma of the next generation with a skewed bimodal distribution in individual cells [108]. In contrast to previously discussed examples of cytoplasmic factor impact on chromatin, only a single locus in this study is (presumably) affected, and how this could affect cytoplasm composition is unclear. As a result, it is hard to fathom how this inheritance could be mediated without a non-DNA methylation germline signal on chromatin, which must somehow escape post-fertilization erasure. Such a distribution can also only be reasoned to be the result of transduction from a continuous signal in germ cells to DNA methylation in the soma.

There have also been other hints of possible transduction from continuous germline chromatin signals to DNA methylation in recent years. The retention of some marks on the *A*^*vy*^ locus specifying its oocyte methylation status is supported by the observation that its silencing in mouse embryonic stem cells is independent of DNA methylation [109]. *In vivo*, DNA methylation independent imprinting requires a switch from H3K27me3 to DNA methylation post-implantation for its maintenance [110,111]. This phenomenon is also biologically relevant, with maternal deletion of some of the subject genes shown to complement aspects of phenotypes associated with somatic cell nuclear transfer and maternal knockout of *Eed* [112,113]. Whether these genes individually lead to dosage-sensitive phenotypes analogous to DNA methylation-dependent imprints, and how useful these will be in mechanistic experiments, however, remains unknown.

Recognizing the molecular networks responsible for the intergenerational distribution of heritable epigenetic marks on chromatin, or within cytoplasm, during ontogenesis might be crucial for improving the efficiency of producing mammalian conceptuses by advanced assisted reproductive technologies (ARTs), including somatic cell cloning [114] and *in* *vitro* fertilization, either via co-incubation of oocytes with motile spermatozoa [115,116] or via intraooplasmic sperm microinjection [117–119].

## Article highlights

- Different restrictions apply to mediators of epigenetic inheritance that persists over different numbers of generations and through different germline sexes. We focus on maternal intergenerational effects in mammals.
- Epigenetically heritable phenotypes can be stratified based on tractability in experimental systems.
- Only imprinting has been demonstrated to require oocyte chromatin marks.
- All strata show common *trans* mediators of the chromatin state post-fertilization.
- Genes commonly regulated by different strata are done so by different mechanisms in each strata.
- Some non-imprinted epigenetic inheritance is hard to explain without effects on germline chromatin that are somehow transduced through reprogramming and DNA methylation independent imprinting shows this is conceptually possible.
